# Whole-genome DNA similarity and population structure of *Plasmodiophora brassicae* strains from Canada

**DOI:** 10.1186/s12864-019-6118-y

**Published:** 2019-10-16

**Authors:** Afsaneh Sedaghatkish, Bruce D. Gossen, Fengqun Yu, Davoud Torkamaneh, Mary Ruth McDonald

**Affiliations:** 1 Department of Plant Agriculture, University of Guelph, Guelph, ON N1G 2W1 Canada; 2 Agriculture and Agri-Food Canada, Saskatoon Research and Development Centre, 107 Science Place, Saskatoon, SK S7N 0X2 Canada; 3 Département de Phytologie, Université Laval, Québec City, QC, G1V 0A6 Canada

**Keywords:** Genetic diversity, *Plasmodiophora brassicae*, Clubroot, DNA sequencing, Population genetics, Balancing selection, Host shift

## Abstract

**Background:**

Clubroot is an important disease of brassica crops world-wide. The causal agent, *Plasmodiophora brassicae,* has been present in Canada for over a century but was first identified on canola (*Brassica napus*) in Alberta, Canada in 2003. Genetic resistance to clubroot in an adapted canola cultivar has been available since 2009, but resistance breakdown was detected in 2013 and new pathotypes are increasing rapidly. Information on genetic similarity among pathogen populations across Canada could be useful in estimating the genetic variation in pathogen populations, predicting the effect of subsequent selection pressure on changes in the pathogen population over time, and even in identifying the origin of the initial pathogen introduction to canola in Alberta.

**Results:**

The genomic sequences of 43 strains (34 field collections, 9 single-spore isolates) of *P. brassicae* from Canada, the United States, and China clustered into five clades based on SNP similarity. The strains from Canada separated into four clades, with two containing mostly strains from the Prairies (provinces of Alberta, Saskatchewan, and Manitoba) and two that were mostly from the rest of Canada or the USA. Several strains from China formed a separate clade. More than one pathotype and host were present in all four Canadian clades. The initial pathotypes from canola on the Prairies clustered separately from the pathotypes on canola that could overcome resistance to the initial pathotypes. Similarly, at one site in central Canada where resistance had broken down, about half of the genes differed (based on SNPs) between strains before and after the breakdown.

**Conclusion:**

Clustering based on genome-wide DNA sequencing demonstrated that the initial pathotypes on canola on the Prairies clustered separately from the new virulent pathotypes on the Prairies. Analysis indicated that these ‘new’ pathotypes were likely present in the pathogen population at very low frequency, maintained through balancing selection, and increased rapidly in response to selection from repeated exposure to host resistance.

## Background

Clubroot, caused by *Plasmodiophora brassicae* Woronin, is an increasingly important constraint to canola (*Brassica napus* L.) production in Canada [1]. It causes prolific and undifferentiated growth of infected roots, which disrupts uptake of water and nutrients by the plant and can result in premature plant death. Clubroot occurs almost everywhere that brassica crops are grown worldwide [2].

Resistant cultivars are currently the cornerstone of clubroot management for canola in Canada, but new virulent pathotypes have emerged and are spreading rapidly [3]. More than 20 canola cultivars that carry clubroot resistance are available in Canada. Resistance to clubroot disease is controlled by R genes [4, 5], but the source of the resistance is not in the public domain [3]. In addition, the molecular mechanism(s) of clubroot resistance are complex [6], so interpretation of host-pathogen interactions are difficult without sufficient information about the genetic background. Pathotypes of clubroot have been characterize based on their ability to cause disease on differential hosts. The most widely used differential sets in North America and Europe include the Williams’ differential set [7], the European Clubroot Differential set [8], the Somé set [9], and most recently, the Canadian Clubroot Differential set [3]. Molecular markers have been identified to distinguish the predominant pathotypes (P11, P9, and P4) in China [10, 11] and pathotype 5X, one of the virulent new pathotypes from canola in Canada [12], but such markers are not yet in widespread use and no markers exist for the other pathotypes.

Genome diversity in plant pathogen populations is the result of migration, random genetic drift, mutation, recombination, and natural selection [13]. *Plasmodiophora brassicae* is a soil-borne pathogen, so migration and gene flow among regional populations almost certainly occurs most commonly as the result of human activities, although movement via wind and water also contribute to pathogen dispersal [1].

There are numerous reports and illustrations of clubroot in the 16th and 17th centuries from Europe [2]. Clubroot was likely brought to North America from Europe in contaminated animal fodder [14]. In Canada, clubroot was initially reported on brassica vegetables in British Columbia, Alberta, Quebec and the Maritimes in the 1920s [15]. Canola is a multi-billion dollar crop [1] on the Canadian Prairies (provinces of Alberta, Saskatchewan and Manitoba), but clubroot was not detected on canola in this region until found in Alberta in 2003 [15]. The pathogen is spreading quickly on canola, and 2443 fields in Alberta had been confirmed infested by clubroot by 2016. The source of the initial infection on canola has never been determined. Following its initial identification in Alberta, clubroot on canola was identified in Manitoba in 2009 [16], in Saskatchewan in 2010 [17], and in Ontario in 2016 [18].

In the United States, clubroot was first reported around New York in 1853 [19] and was identified on canola for the first time in North Dakota in 2013 [20]. Recent surveys demonstrated a 5-fold increase in clubroot infested canola fields in North Dakota from 2017 to 2018 [21]. In contrast to the rapid spread of clubroot on the Prairies and in North Dakota, clubroot is spreading much more slowly in central Canada, likely due to smaller amounts of inoculum and small, dispersed acreages of susceptible hosts [1].

China is also a major producer of brassica crops and clubroot is becoming a constraint to production in many regions. Clubroot was first reported in Taiwan in 1910 [22] and in Fujian province in 1947 [23]. An outbreak of clubroot was reported in several southern provinces of China, including Jiangsu and Yunnan in 1962 [23]. By the 1990s, clubroot was reported in southern, northern, and central regions [23]. Recently, clubroot was detected on canola in Anhui, Sichuan, and Hubei provinces [23]. Clubroot was initially reported in both Canada and China in early nineteenth century on brassica vegetables and has spread to canola in recent years in both countries. However, the most common hosts of *P. brassicae* in China are brassica vegetables, while in Canada the most common host is canola. Comparison of genetic similarity in the pathogen population in China relative to the population in Canada might provide insights into the direction of selection in response to different hosts and environments.

The life cycle of *P. brassicae* involves two main stages: a primary stage in which root hairs are infected by primary zoospores produced by long-lived resting spores in soil, and a second stage where secondary zoospores from root hairs infect and colonize the root to produce resting spores [24]. Mitotic divisions occur in the primary stage, when primary plasmodia (multi-nucleate) divide to produce secondary zoospores, and in the second phase when secondary plasmodia (also multi-nucleate) divide to produce resting spores [25]. Fusion of secondary zoospores and subsequent meiotic division to produce secondary plasmodia has been postulated [24, 26] but has not been confirmed.

There are relatively few genomic studies of *P. brassicae* because obtaining clean DNA of this obligate, intracellular pathogen is difficult [27]. DNA from resting spores in clubbed roots is invariably accompanied by DNA from the host, and often contaminated by DNA from saprophytic microbes. In addition, *P. brassicae* may be present as a mixture of pathotypes within a single infected root [27]. Although a few isolates of *P. brassicae* have been sequenced to date [28–30], study of the genomics and population genetics of *P. brassicae* is still in its infancy. Analysis of the single-spore isolate e3 from Europe estimated the total genome size of *P. brassicae* to be 25.5 Mb [27]. In a study of the genome sequence of pathotypes 3 and 6, the total size of each genome was 24.2 Mb [27]. When the genomes of six single-spore isolates of pathotypes from Canada were sequenced, pathotypes 2 and 3 were similar, pathotypes 5 and 8 were similar, and pathotype 6 differed from the other isolates but was similar to e3 from Europe [29]. In another study, a field collection of pathotype 6 from vegetables in British Columbia differed substantially from collections from canola in Alberta [30].

Although *P. brassicae* cannot be grown in pure culture, dual tissue cultures of *P. brassicae* with a host species have been used to culture this pathogen. Dual cultures of *P. brassicae* with callus of *Brassica* spp. have been used to observe the development of *P. brassicae* within host cells with minimal contamination by soil microbes [24, 31–35] and has been used to provide pure samples of DNA for genomic analysis of other plasmophoroids [34, 36].

The main objectives of the study were to compare the genomic structure of *P. brassicae* populations from different locations and hosts to assess the genetic variation present in the pathogen population across Canada, and if possible, to obtain insights into the origin of the original source of infection on canola in Alberta. Also, obtaining whole-genome sequences of many *P. brassicae* populations might provide insights into the rapid emergence of new pathotypes on canola in Canada, which would improve breeding efforts to manage clubroot and potentially accelerate the development of molecular markers to identify specific pathotypes.

## Results

### Dual callus cultures

Dual callus cultures of *P. brassicae* with *B. rapa* were established on Murashiga and Skoog (MS) medium for about 50% of the strains assessed. White callus cells grew from the edges of the root tissue after 2 weeks. Only clean (non-contaminated) cultures were transferred to fresh media. After two transfers, aliquots of callus cells were cut from the root pieces and transferred to MS media without antibiotics. In general, callus formed more frequently from roots harvested 4 weeks after inoculation compared with 6 weeks. Twenty of the 52 *P. brassicae* strains produced clean calli. Multiple stages of the *P. brassicae* life cycle were observed in calli.

Hypocotyl, cotyledons, leaves, and roots of *B. rapa* were tested for their potential to produce callus in the absence of *P. brassicae*, to identify a source of materials that could be used as a noninfected control. Calli were small, formed infrequently, only formed from *B. rapa* root cultures, and generally developed in response to wounding.

### DNA variants

**Fig. 1:**
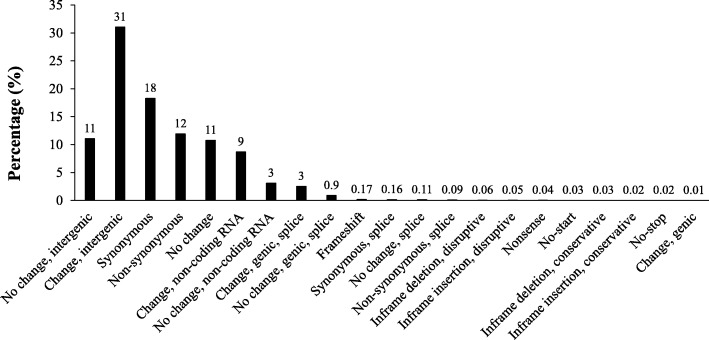
DNA variant distribution (%) in genic and intergenic regions of 43 strains of *Plasmodiophora brassicae*

Nine samples that did not meet the requirement for minimum template coverage of 90% per strain were not included in the analysis. The mean template coverage of included strains was 91.9% per isolate. In total 149,774 variants were identified, with 142,640 SNPs and 6218 Indels. Out of 149,774 variants, 145,284 variants were polymorphic within this population. The variant type and distribution were as follow: 58% of the variants identified in genic regions occurred in coding regions (exons) and 42% in non-coding regions (introns); 58% of variants were transition, 36% transversion, 3% deletion, and 3% inversion types. Also, 18% of variants were synonymous, while 12% were non-synonymous (Fig. 1). Variants were more common in intergenic regions (> 40%) than in genic regions (< 5%). In 42 of 43 strains, more than 75% of the SNPs were homozygous (Additional file 1: Figure S1), but 25–75% of SNPs were heterozygous in one collection from Alberta (AB9-C-P5).

### Population structure and principal component analysis

**Fig. 2:**
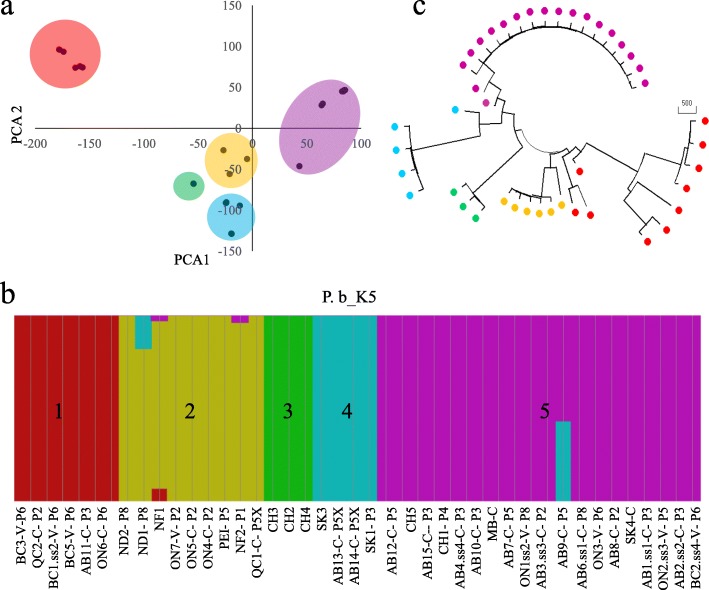
Three assessments of genetic diversity in the sequenced strains of *Plasmodiophora brassicae*, with strains of the same clade represented by the same color: **a** principal component analysis of 43 Canadian strains; **b** population structure analysis of all 43 strains, where each vertical column represents one strain, each color represents a clade (1–5) and admixtures are represented by two colors, and (**c**) a neighbor-joining tree of the 43 strains

Principal component analysis (Fig. 2a) and population-structure analysis (Fig. 2b) of the 43 *P. brassicae* strains were used to determine the composition of the population and the number of the clades. An optimal K value of 5 (describes the number of clades that make up the total population) explained the structure in the data (Fig. 2c). Population-structure analysis indicated that strains AB9-C-P5 from Alberta, NF2-P1 and NF1 from Newfoundland, and ND1-V-P8 from North Dakota were admixtures of two genotypes.

### Phylogenetic tree

**Fig. 3:**
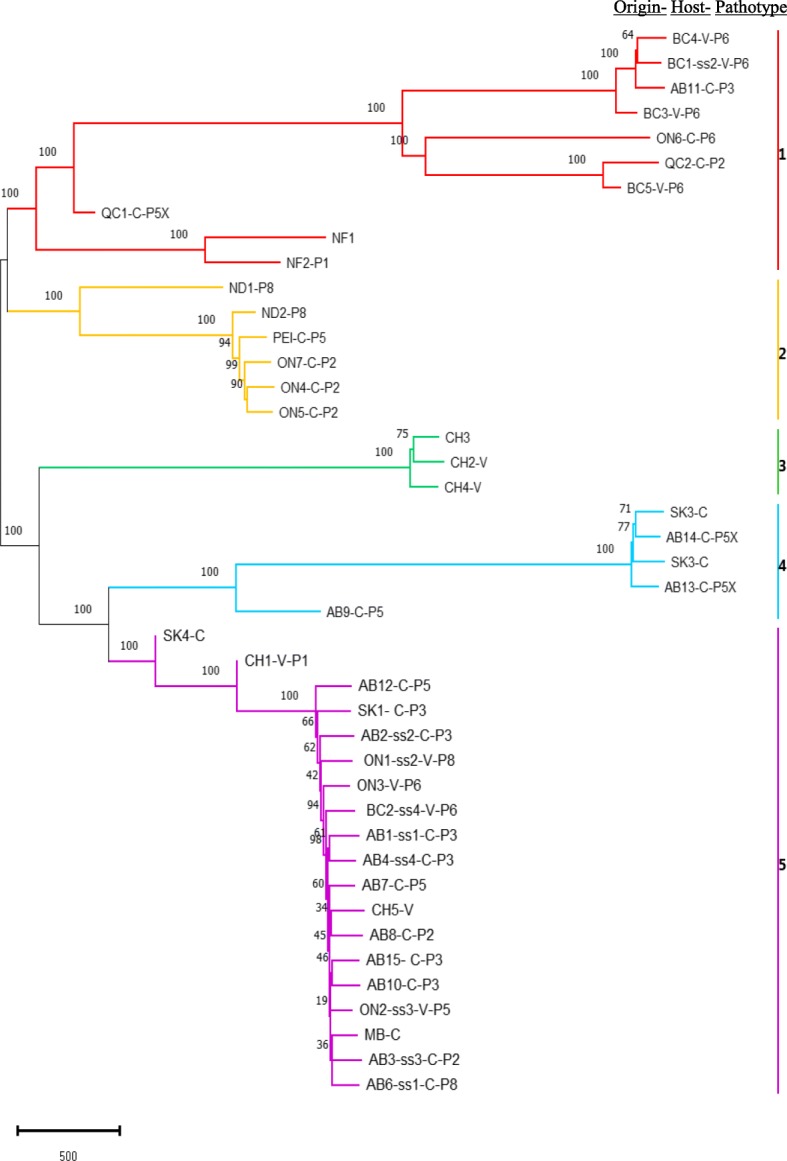
SNP-based phylogenetic tree of *Plasmodiophora brassicae* based on whole-genome alignment of 43 *Plasmodiophora brassicae* strains from Canada, China, and the USA mapped against e3 from Europe. The scale represents the evolutionary distances. The percentage of 1000 bootstrap trials (1000 replicates) are shown above the branches. Strains details are summarized in their names: location by province (e.g., AB = Alberta), Williams’ pathotype (e.g., P3) where available, and SS = single-spore isolate, V = vegetable host, and C = canola host

Phylogenetic trees were constructed using three clustering methods; neighbor joining (Fig. 3), maximum likelihood (Additional file 2: Figure S2), and Euclidean hierarchical distance (Additional file 3: Figure S3). The trees produced by all three methods were very similar and would lead to the same conclusions. Only neighbor joining tree was used for further analysis, because the neighbor joining method is optimal for big data sets compared with the maximum likelihood, which is more accurate for very small data sets. In addition, the neighbor joining method is the most commonly used distance method to compute *P. brassicae* phylogenetic trees [29, 30]. The neighbor joining tree was developed using genome-wide SNPs (total = 10 K SNPs) of 43 strains, which grouped the strains into five clades (Fig. 3). This result was consistent with separation into five clades based on the population structure and PCA (Fig. 2). The proportion (%) of 1000 bootstrap pseudo-replicates that supported each joining is shown above each node [37, 38]. The strains from Canada clustered into Clades 1, 2, 4 and 5.

Clade 1 consisted of the strains from British Colombia, Newfoundland, Ontario, and Quebec, mainly from vegetable brassicas (Fig. 3). Interestingly, a pathotype 3 collection from Alberta in 2008 (predominant pathotype in Alberta, collected before CR cultivars were commercially available) was also allocated to Clade 1 (Fig. 3). Clade 2 consisted mainly of collections from canola in Prince Edward Island, Ontario [18], and North Dakota, USA, from sites where the pathotype has changed. Three of the five strains from China clustered together in Clade 3. Strains from the Prairies mostly clustered into Clades 4 and 5. Clade 4 consisted of the new, virulent pathotypes from Alberta (AB13-C-P5X and AB14-C-P5X), a single recent collection (SK3-C) from Saskatchewan, and collection AB9-C-P5 from Alberta. Most of the strains from canola in Saskatchewan and Manitoba, which represent a recent expansion of the geographic distribution of the pathogen, clustered together with the older strains from canola in Alberta in Clade 5, along with strains from vegetables in Ontario and British Columbia. Two collections from China (one each from Jangsu and Hubei province) also clustered in Clade 5. (Fig. 3).

Within Clade 5, the sequences of single-spore isolate ON1-ss2-V-P8 and its original source collection ON3-V-P6 were almost identical, so they were immediate neighbors in the phylogenetic tree. Similarly, single-spore isolates AB1-ss1-C-P3, AB2-ss2-C-P3, AB3-ss3-C-P2, AB4-ss4-C-P3, AB6-ss1-C-P8 and ON2-ss3-V-P5 differed by only 1.5–3% in SNPs from their source collection, AB15-C-P3 and AB7-C-P5.

**Fig. 4:**
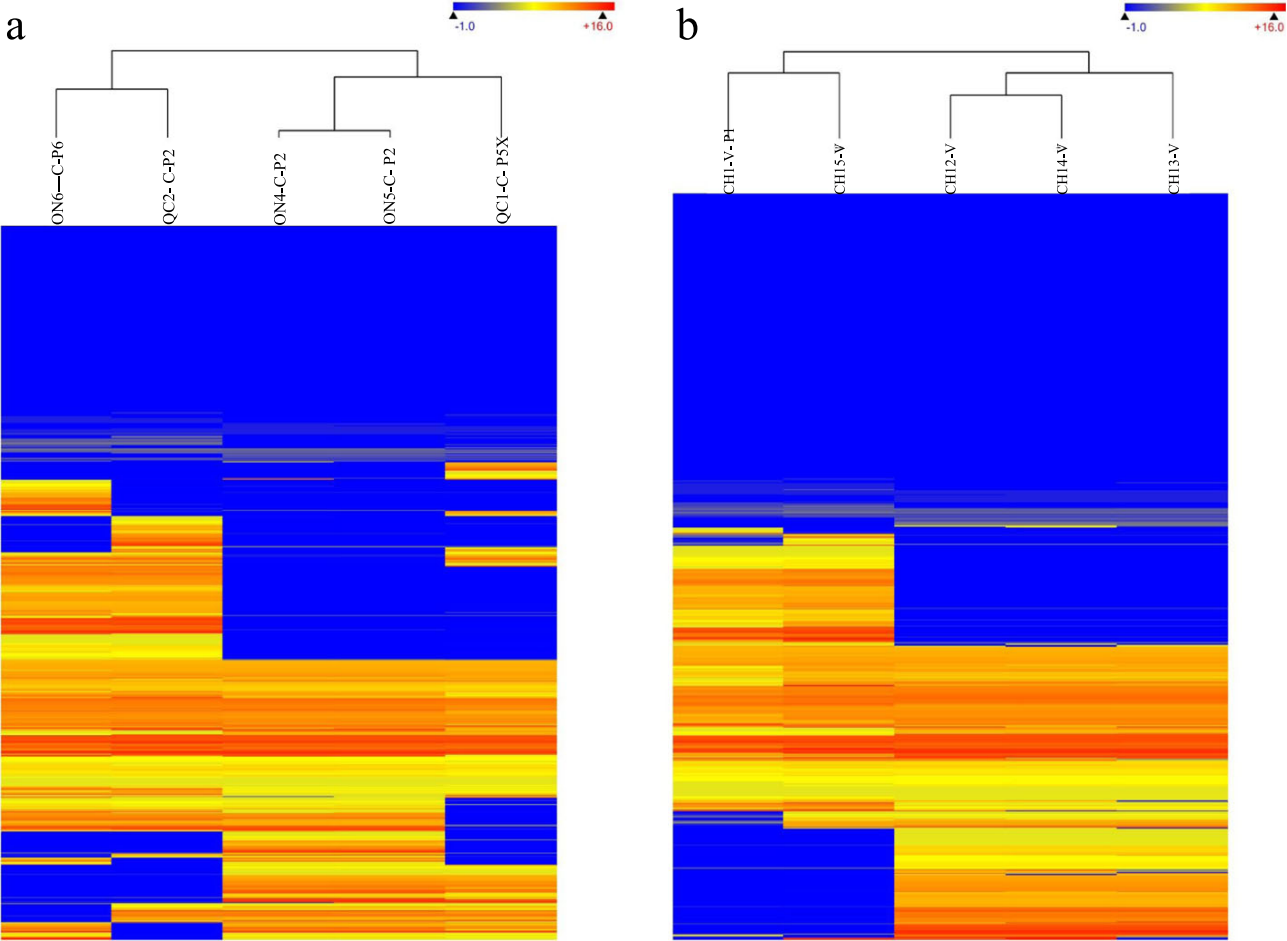
Heat maps of SNPs distribution in *Plasmodiophora brassicae*. Strains from (**a**) Normandin and MCRS before and after the change of pathotype, and (**b**) Chinese strains were compared. Each horizontal lane represents a gene. The total gene number in the genome is 9727

At the AAFC research farm at Normandin QC, genetic resistance has broken down and pathotype 2 has been replaced by pathotype 5X. Similarly, at the Muck Crops Research Station in Ontario, pathotype 6 has been replaced by pathotype 2. The initial collections clustered with Clade 1 and samples collected after the change of pathotype clustered in Clade 2. Samples that has been collected before and after the breakdown in resistance and associated change of pathotype from the two sites [39] were compared using heat map analysis. SNP polymorphisms were present in more than half of the total 9727 genes in the genome between samples collected before and after the change of pathotype at each site (Fig. 4a). Similarly, SNP polymorphisms were present in more than 3000 genes between the Chinese collections from Clade 3 (CH2-V, 3 and 4) and the Chinese collections from Clade 5 (CH1- P1-V 4 and CH5-V) (Fig. 4b).

DNA samples extracted from both a callus and a frozen club of SK3-C were sequenced and compared to assess the reproducibility of the sequencing. The two samples of SK3-C clustered in the same clade, with only 3% difference in gene similarity between the two samples. This difference may have been associated with trace amounts of microbial contamination in the sample taken directly from the clubbed root, or inherent variability among strains of the pathogen in a single club.

Collection BC3-V-P6 and single-spore isolate BC1-ss2-V-P6 from British Columbia clustered with the other strains from vegetable crops in British Columbia in Clade 1. However, single-spore isolate BC2-ss4-V-P6 clustered with strains from canola on the Prairies in Clade 5. The same pattern was observed in samples from Ontario. Single-spore isolates ON1-ss2-V-P8 and ON2-ss3-V-P5 from Ontario differed from their source collection, ON3-V- P6, and clustered with strains from the Prairies in Clade 5, while other strains from Ontario clustered in Clades 1 or 2. These two single-spore isolates were also different pathotypes from the source collection.

### Genome diversity

Genome-wide nucleotide diversity was calculated by examining DNA sequences to quantify the degree of polymorphism in a population at a nucleotide level, and was used to determine the DNA divergence between subpopulations. Nucleotide diversity is the average number of pairwise nucleotide differences between sequences and depends on the number of polymorphic sites and their relative frequencies. Genome-wide nucleotide diversity was estimated based on sliding windows of 1 kb across the 43 strains. The mean nucleotide diversity (θπ) was higher in Clades 1 and 3 (θπ1 = 0.0011, θπ3 = 0.0011) compared with Clades 2, 4 and 5 (θπ2 = 0.00097, θπ4 = 0.00084, and θπ5 = 0.00084). The average θπ across the whole genome of all strains was 0.00095 (Additional files 4 and 5). When the evolutionary distance between clades was computed, the distance based on the length of the branch was largest between Clades 1 (Canadian collections from vegetable brassicas) and 3 (collections from China), and smallest between strains from the Prairies (Clades 4 and 5) and Clade 3.

**Fig. 5:**
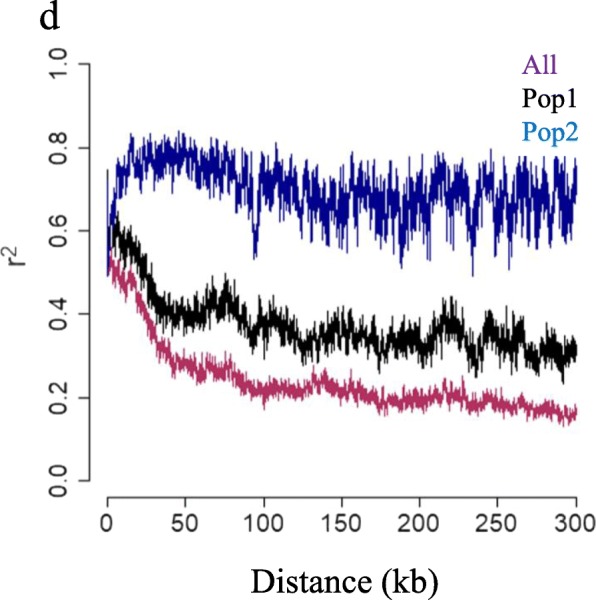
Linkage disequilibrium decay (*r*^*2*^) versus the distance between polymorphic sites in Pop 1 (Clades 1 and 2) and in Pop 2 (Clades 4 and 5) of 43 strains of *Plasmodiophora brassicae*

Linkage disequilibrium (the non-random association of alleles at different loci, LD) between two loci decays gradually in proportion to the recombination rate and time over the generations. Linkage disequilibrium decay was measured by determining the distance between polymorphic sites at which the LD is halved when using the 50% the squared allele frequency correlation (r^2^) as estimator for LD decay. The average distance over which LD decayed to half of its maximum was at ~ 29 kb. The decrease of linkage disequilibrium was slower in strains from the Prairies compared with strains from the rest of Canada in the 43 strains (Fig. 5).

The capacity for populations to sustain variation depends on the population size and the mutation rate. The amount of variation that a population carries can be mathematically calculated from genome sequences. If a deviation from the expected variation is observed, it indicates that a particular form of selection is present. Tajima’s test of neutrality (D) detects selection from within population polymorphism. Tajima’s D value can be positive (the observed variation was higher than the expected level) or negative (the observed variation was lower than expected). Tajima’s D value was 0.73 when calculated across the 43 *P. brassicae* populations. A positive value for D indicates balancing selection, while a negative value indicates purifying selection or a recent population expansion.

## Discussion

In the current study, the genetic similarity of *P. brassicae* strains from across Canada and selected locations in China and the USA were assessed using whole-genome DNA sequences and compared with a published genome sequence from Europe. The limited number of single-spore isolates assessed were often nearly identical to the collections they were taken from. A phylogenetic tree that consisted of five clades was developed based on SNP polymorphisms and validated using several approaches.

Strains of *P. brassicae* from vegetables across North America formed Clade 1. Clade 2 was mainly from canola outside of the Canadian Prairies at sites where the change in pathotype had been detected. Three of five collections from China clustered together in Clade 3, which was substantially different from strains in North America and the single-spore isolate from Europe. Strains from canola on the Prairies mostly clustered into Clades 4 and 5. Clade 4 consisted of the new, virulent pathotypes from Alberta. Most of the strains from canola in Saskatchewan and Manitoba, which represent a recent expansion of the geographic distribution of the pathogen, clustered together with the older collections from canola in Alberta and two collections from China in Clade 5.

Clades 1 and 2 clustered separately from the initial strains from canola on the Canadian Prairies (Clade 5), but differed even more from the cluster of new, virulent pathotypes that are currently increasing in the Prairie region (Clade 4). A separate clade consisting of collections from China was expected, given the relatively long period of geographic separation and differential selection between the pathogen populations in North America and China, but the observation that 2 of 5 collections from China clustered with the original strains from canola in Alberta indicated that selection pressure may be driving both populations in a similar direction.

In the current study, collections AB13-C-P5X and AB14-C-P5X (new pathotype 5X from Alberta) were very different from the older collections from Alberta, with SNP disruption of function in thousands of genes. It is unlikely that this new, virulent pathotype could have accumulated thousands of mutations since the initial registration of a clubroot-resistant canola cultivar in 2009. However, the possibility of mixed populations of pathotypes being present within a club might have influenced the gene diversity among field collections.

Similarly, genetic diversity was high between collections taken before and after a change of pathotype at sites in Quebec and Ontario. The high diversity between these collections indicated that the likelihood that the new pathotypes developed from the original pathotypes was very low. These results indicated that the ‘new’ pathotypes were the result of selection of pre-existing genotypes present at low levels in the population (rather than from mutation to virulence) that increased rapidly in response to selection first on a new host and later on resistant cultivars.

Population-structure analysis was also used to identify the migrants between two populations. Population-structure analysis indicated that collections AB9-C-P5 from Alberta, NF2-P1 and NF1 from Newfoundland, and ND1-V-P8 from North Dakota were admixtures of two populations. Admixtures are generally the result of directional selection due to migration and isolation [40]. NF2-P1 and NF1 strains collected from Newfoundland were found in Clade 2 but were also occasionally assigned to Clades 1 or 5. Ab9-C-P5 was an admixture of Clades 4 and 5, while ND1-V-P8 from North Dakota was an admixture of Clades 2 and 4.

Nucleotide diversity (π) depends on factors such as population size, mutation rate, and reproduction states [40]. A previous study estimated nucleotide diversity per site of 0.32 for *P. brassicae* strains in Alberta [30]. In the current study, diversity was high between strains from the Prairies and the rest of Canada; for the whole genome, θπ = 0.0009, and for average diversity per site, π = 0.22.

Sexual recombination is another important reason for genetic variation, which shapes the impact of selection in the genome. The timing and occurrence of sexual reproduction, if it occurs at all in the life cycle of *P. brassicae*, is not well understood [41]. It has been suggested that secondary zoospores may fuse before the cortical infection phase [42, 43], but no such fusion was observed in a recent study of secondary zoospores [41]. Sexual reproduction between different genotypes is clearly not an absolute requirement for infection by *P. brassicae*, since inoculation of a host with a single resting spore can result in infection and symptom development [43]. Also, no sexual recombination was detected in a recent study with repetitive molecular probes [44]. However, non-meiotic recombination processes such as gene conversion and mitotic crossing-over have been reported in *P. brassicae* [44].

To gain more knowledge about the occurrence of recombination in *P. brassicae*, linkage disequilibrium was calculated. Linkage disequilibrium generally represents a pronounced deviation from the random pattern associated with sexual reproduction [13]. Linkage disequilibrium can be also caused by migration, genetic drift, selection, or other factors [45]. Linkage disequilibrium between collections from the Prairies and the rest of Canada was assessed to better understand the genetic forces contributing to the development of new pathotypes (Fig. 5, Additional file 6). Disequilibrium was higher in strains from the Prairies relative to strains from the rest of Canada. This demonstrated that fewer genetic changes had occurred in Prairie populations compared with those in the other provinces in Canada.

The rate that linkage disequilibrium decays with time over generations is influenced by the recombination frequency. In self-fertile plants, linkage disequilibrium decays more slowly and at a large genetic distance (up to 20 cM) [46] than in outcrossing species. In contrast, decay occurs faster (within 100–1500 bp) in a heterozygous background with several recombination events [47]. One approach used to estimate linkage disequilibrium decay is to find the distance at which half of the maximum linkage disequilibrium has decayed [48, 49]. The half decay of disequilibrium occurred at 29 kb when measured across all 43 *P. brassicae* strains. This value was in the range that has been reported for 10 fungal species, in which the half decay value was 110 bp for species with a mixed reproductive mode and > 100 kb for clonal species [50]. Relatively rapid decay of linkage disequilibrium in *P. brassicae* populations indicated a high frequency of recombination in the population, which in turn supports the hypothesis that *P. brassicae* is not a clonal species.

Tajima’s test of neutrality was performed to determine if balancing selection was apparent in the *P. brassicae* strains. When balancing selection is present, populations maintain variation, so alleles with low frequency or that are less fit are not removed from the population [51–53]. Balancing selection might help the population adapt to a sudden change, such as exposure to a new host. Tajima’s D was 0.73 for 43 *P. brassicae* strains. The positive value of D indicated that balancing selection was present in these populations. This supported the observations of several previous studies [3, 54, 55], which reported a high percentage of diversity and the preservation of rare pathotypes in *P. brassicae* populations. The unique infection process of *P. brassicae*, which starts with thousands or millions of separate infections of root hairs from individual resting spores that, collectively result in production of one or a few large clubs, appears to be suited to result in balancing selection.

At present, no information is available on the mutation rate of *P. brassicae.* Random genetic drift is unlikely to result in rapid genome variation of *P. brassicae* because of the large populations of long-lived resting spores in soil.

Only a limited number of studies of genetic diversity among regional populations of *P. brassicae* are available, generally based on partial genome sequences rather than the entire genome. Populations of *P. brassicae* were highly diverse among regions of Germany [55], and among fields and even years at a single site in north-western France [54]. In contrast, the diversity was so low among 21 isolates from Alberta that the authors concluded that the population was clonal [30]. None of these studies showed a correlation among host source, virulence or DNA pattern of *P. brassicae* [54, 56], which is consistent with the current study.

In another study, a single-spore isolate from vegetables in British Columbia (ABOT-JE ss1) differed substantially from 20 field collections and single-spore isolates in Alberta [30]. ABOT-JE ss1 was not available for the current study, but the original field collection BC3-V-P6 (ABOT-JE-04-01) and two single-spore isolates from the same initial collection (BC1-ss2-V-P6 and BC2-ss4-V-P6) were included. Field collection BC3-V-P6 and single-spore isolate BC1-ss2-V-P6 were very similar to e3 from Europe, which is in consistent with the previous study [29].

Genetic similarity with e3 from Europe was high for Clades 1 and 4, and much lower for Clades 2 and 5. This same pattern was apparent in the lack of similarity between the pathotypes present before and after resistance breakdown, as illustrated both in the phylogenetic tree and heat map analysis of strains from the same site. Both of these broad patterns support the hypothesis that pathotype 3 had likely been present in the initial population(s) introduced to North America from Europe but was present only at very low levels on brassica vegetables on the Prairies. Pathotype 3 became dominant on canola in Alberta only after repeated exposure (and selection for virulent pathotypes) on a new host, canola. Prior to the release of the first clubroot-resistant canola cultivar in 2009, all canola cultivars on the Prairies were susceptible to pathotype 3. Currently, selection pressure from the widespread use of clubroot-resistant cultivars has resulted in the increase of numerous new pathotypes capable of overcoming the genetic resistance available in the initial generation of clubroot-resistant canola cultivars. The diversity and rapid development of these new, virulent pathotypes [3] further supports this conclusion. It also indicates that there may be many pathotypes still to be found among the quadrillions of resting spores currently present in the soil on the Prairies.

The current study demonstrated that genetic diversity is high in *P. brassicae* populations in Canada. This high diversity may be largely the result of two conflicting forces: balancing selection in *P. brassicae*, which results in the maintenance of a wide variety of genetic lineages in the pathogen population, and selection pressure associated with differences in host and source of genetic resistance. Genetic diversity in *P. brassicae* was lower on the Prairies, where it encounters a narrow host range (primarily canola and susceptible weeds) relative to other production areas where the pathogen encounters a broad range of brassica vegetables.

It is not known when the pathogen was introduced to Alberta, but data from the current study support the hypothesis that pathotypes virulent on canola likely took many years to build up to detectable levels because they occurred at low level in the initial populations introduced to the region on brassica vegetables. In addition, new pathotypes appeared on the Prairies due to selection pressure from the widespread use of resistant canola cultivars. Each clade in the phylogenetic tree contained multiple pathotypes and strains from various hosts, which indicated that pathotype and host were not the principal factors in grouping the strains. Finally, the current study demonstrated that dual tissue cultures provided an excellent source of DNA for sequencing this difficult and unique pathogen.

## Conclusion

Assessments of genetic diversity based on whole-genome sequences indicated that the ‘new’ pathotypes on canola had likely been present in the pathogen population at very low levels, maintained through balancing selection, and became dominant due to selection associated with a host shift. The high linkage of loci in strains from the Prairies should make it possible to identify markers that correlate with pathotype.

## Methods

### Clubroot material and plant inoculation

Samples of the pathogen that have not been converted to single-spore isolates are referred to as a ‘collections’, and single-spore isolates and collections together as strains. Fifty-two strains were obtained from various regions of Canada, the USA and China (Table 1). For materials collected in Canada, those from British Columbia were provided by Dr. S.E. Strelkov (BC1–BC3) at the University of Alberta, Edmonton, AB, and Dr. J. Elmhirst (BC4 and BC5) of Dr. Elmhirst Diagnostics and Research, Abbotsford, BC, those from Alberta were provided by Dr. Strelkov (AB1–AB10, AB13–AB15) and Dr. M.R. McDonald (AB11, AB12) of the University of Guelph, Guelph, ON, from Saskatchewan and Manitoba by Dr. B.D. Gossen (SK1–SK3, MB1) of the Saskatoon Research and Development Centre of Agriculture and Agri-Food Canada at Saskatoon SK, from Ontario and Quebec by Dr. M.R. McDonald (ON1–ON6, QC1, QC2), from PEI by Dr. S.H. De Boer (PEI1) of the Canadian Food Inspection Agency in Charlottetown, PEI (retired), and from Newfoundland by Dr. L. Jewel (NF1, NF2) of Agriculture and Agri-Food Canada in St. Johns, NL. Isolates from North Dakota USA were provided by Dr. L. Del Rios (ND1, ND2) of North Dakota State University, Fargo, ND, and the isolates from China (Hebei, Yunnan, and Jiangsu provinces, representing a range of pathotypes collected from brassica vegetables) were provided by Dr. Jianbin Li (CH1–CH5) at the Institute of Vegetable Crops, Jiangsu Academy of Agricultural Science.

**Table 1:** Origin, names, pathotype (Williams’s system) and source of field collections and single-spore isolates (SS) of *Plasmodiophora brassicae* used for whole-genome sequencing

Origin	Name	Year	Original designation	Source and Reference
Canada				
BC	BC1-ss2-P6		AbotJE	Napa cabbage [57]
BC	BC2-ss4-P6		AbotJE	Napa cabbage [57]
BC	BC3-P6		AbotJE-04-01	Napa cabbage [57]
BC	BC4-P6	2015	P6	Brussels sprouts
BC	BC5-P6	2017	P6	Cauliflower
AB	AB1-ss1-P3		SACAN	Canola [57]
AB	AB2-ss2-P3		SACAN	Canola [57]
AB	AB3-ss3-P2		SACAN	Canola [57]
AB	AB4-ss4-P3		SACAN	Canola [57]
AB	AB6-ss1-P8		CDCN	Canola [57]
AB	AB7		CDCN-04-01	Canola [57]
AB	AB8-P2	2005	F.1–05	Canola [58]
AB	AB9	2005	F.290–07	Canola [59]
AB	AB10-P3	2017	P3	Canola
AB	AB11-P3		Deora	Canola [60]
AB	AB12-P5		Deora	Canola [60]
AB	AB13-P5X		LG1	Canola [61]
AB	AB14-P5X		LG3	Canola [61]
AB	AB15-P3		SCAN.03.01	Canola [57]
SK	SK1-P3	2017	Aug25	Canola
SK	SK2	2017	CD1A	Canola
SK	SK3	2017	Sep 21	Canola
MB	MB	2017		Canola
ON, Orton	ON1-ss2-P8		ORCA	Cabbage [57]
ON, Orton	ON2-ss3-P5		ORCA	Cabbage [57]
ON, Orton	ON3		ORCA-04-01	Cabbage [57]
ON, MCRS	ON4-P6	2016	P6	Canola [39]
ON, MCRS	ON5-P2	2017	P2	Canola [39]
ON, MCRS	ON6-P6	2011	P6	Canola [39]
ON, Verner	ON7-P2		P2	Bok choi [18]
QC, Normandin	QC1-P5X	2017	P5X	Canola [39]
QC, Normandin	QC2-P2	2012	P2	Canola [59]
PEI	PEI1-P5	2017	P5	Canola
NF	NF1	2016	DD1	Vegetable
NF	NF2-P1	2016	DD2A	Vegetable
USA				
ND	ND1-P8		NDCR1	
ND	ND2-P8		NDCR4	
China (CH)				
Jiangsu, Ganyu	CH1-P1		4	Gailan
Yunnan, Muding	CH2		8	Chinese cabbage
Yunnan, Muding	CH3		11	Chinese cabbage
Yunnan, Lufong	CH4		12	Cabbage
Hebei, Kuyuang	CH5		28	Broccoli

*BC* British Colombia, *AB* Alberta, *SK* Saskatchewan, *MB* Manitoba, *ON* Ontario, *QC* Quebec, *NF* Newfoundland, *PEI* Prince Edward Island, *ND* North Dakota, *CH* China, *MCRS* Muck Crops Research Station

The strains supplied by Dr. Strelkov were provided under regulation by the Government of Alberta, and cannot be transferred without permission of that regulator. Similarly, the strains from the United States and China were imported under the regulations of the Canadian Food Inspection Agency of the government of Canada for use in the PPC-2 facility at the Agriculture and Agri-Food Canada Centre at Saskatoon and cannot be distributed without permission of that regulator. The other strains were provided without constraint to distribution.

Three of five single-spore isolates from a previous Canadian study [29] were included in the current study, but the DNA sequences from that previous study were not yet available in GenBank when the sequences for the current study were analysed.

To increase inoculum for the study, seed of Shanghai pak choy (*B. rapa* var. *chinensis*) cv. Mei Quing Choi (Stokes Seeds, St. Catharines, ON, Canada) was planted and grown for 7 days in Sunshine #4 soilless mix (Sun Gro Horticulture, MA, USA) in tall plastic pots (Stuewe and Sons Inc., OR, USA), with two seeds per pot and seven pots per replicate. The plants were maintained at 25°/20 °C day/night, with 16-h photoperiod and 65% relative humidity, and thinned to one seedling per pot. Each 7-day-old seedling was inoculated at the base of the stem with 5 mL of resting spore suspension (10^6^ to 10^8^ spores mL^− 1^) of selected strains following the method of Sharma et al. [62]. Controls were inoculated with 5 mL water. Four to six weeks after inoculation, the plants were uprooted, and clubbed root samples were taken for either tissue culture propagation or direct DNA extraction.

### Dual callus cultures

The infected roots from 4- to 6-week-old inoculated plants were washed and surface sterilized as follows: the outer layer of each root was trimmed off, then a short length of trimmed root was surface-sterilized with 95% ethanol for 1 min, 30% commercial bleach for 20 min, and 2% chloramine T biocide for 10 min. Each root piece was then washed three times with sterile deionized water [34] and cut aseptically into thin cross-sectional pieces. Each piece was placed individually in 90-mm-dia. Petri dishes containing 5 mL Murashige and Skoog (MS) medium (Murashige & Skoog, 1962) including Gamborg vitamins [63] (Sigma Aldrich, Mississauga, ON, Canada) plus 3% sucrose, 300 mg. L^− 1^ Timentin (Thomas Scientific, Swedesboro, NJ, USA), and 0.8% Gelrite (Sigma Aldrich). The infected root pieces were incubated for 3–4 weeks at 21 °C in darkness. Where *Plasmodiophora-*induced callus cultures developed, they were maintained by transfer to fresh medium every 2 weeks. After two transfer cycles (callus > 0.5 cm), the callus was cut from the infected root pieces and transferred to MS media that did not contain antibiotic. At the end of this last cycle, each culture was assessed for contamination using a light microscope and only clean cultures were transferred to fresh medium.

To produce a callus control in the absence of *P. brassicae*., seeds of *B. rapa* were disinfected in 10% household bleach for 10 min, rinsed several times in sterile water and placed on filter paper in sterile Petri dishes at room temperature for 6 days. Hypocotyl, cotyledons, leaves, and roots of 6-day-old seedlings were cut into 5-mm pieces and placed on MS media containing Gamborg vitamins to produce callus cells.

### DNA extraction

Genomic DNA was extracted from 100 mg of freeze-dried club or 100 mg of callus tissue. Clubs were disinfected with 30% household bleach for 10 min, 90% ethanol for 1 min and 2% chloramine T for 20 min prior to freeze-drying for 72 h. DNA was extracted using a modified CTAB method, which was selected because it resulted in higher concentrations of pathogen DNA than other methods in preliminary assessments. The tissue was homogenized for 3 × 1 min at 20 revolutions s^− 1^ using a Qiagen Tissue lyser II with MP BIOMEDICALS Lysing Matrix A tubes. Homogenized tissue was resuspended in 500 μL cetyltrimethylammonium bromide (CTAB) buffer (2 M Tris pH 8.0, 0.5 M EDTA, 5 M NaCl and 2% CTAB) plus 2 μL of 2-mercaptoethanol (Sigma Aldrich) for each sample and incubated at 65 °C for 1 h. The solution was extracted once with 500 μL chloroform and precipitated in 250 μL of 2-propanol. DNA was resuspended in water and treated with RNase. Extracted DNA was quantified using a Qubit fluorometer (Life Technologies, Eugene, OR).

### DNA sequencing and identification of DNA variants

Genomic libraries and DNA sequencing were carried out at the National Research Council of Canada, Saskatoon, SK using Illumina HiSeq 2500 paired-end read technology (2 × 125 cycles, average 200 million reads). *Plasmodiophora brassicae* reads were identified by mapping the DNA-seq libraries to the e3 reference genome of *P. brassicae* (assembly GCA_001049375.1) using the SeqMan NGen assembly program of the Lasergene 15 software package (DNAStar, Madison, WI). The ArrayStar program of the Lasergene package was used for analysis of single nucleotide polymorphisms (SNPs). Assembly and variant parameters were as follows: mer size, 21; minimum match, 93%; minimum read depth, 5; P not ref (fixed), 10; minimum variant count, 2; minimum base quality score, 15; alignments cut-off, 200; maximum gap size, 6; minimum aligned length, 35; match score, 10; mismatch penalty, 20; gap penalty, 50; gap extension penalty, 5; auto trim reads, true; variant detection mode, haploid; minimum variant 15. In total, 43 isolates (Table 1) and about 10,500 SNPs were used in the subsequent phylogenetic analyses (Additional file 7). Data filtering was performed using VCFtools [64]. Variants were removed if: i) they had more than two alleles (this removes a high frequency of genotyping errors, and is also required for subsequent analysis of factors such as structure), ii) an allele was not supported by reads on both strands, iii) the overall quality (QUAL) score was < 32, or iv) the mapping quality (MQ) score was < 30.

### Population structure and principal component analysis

The population structure, based on genome wide SNPs, was determined using the STRUCTURE program. Population structure was estimated using the variational Bayesian inference software in fastSTRUCTURE [65]. Ten runs were performed for each number of populations (K) set from 1 to 12. The most likely K value was determined by the log probability of the data (LnP(D)) and delta K, based on the rate of change in LnP(D) between successive K values, where model complexity that maximized marginal likelihood = 8 and model components used to explain structure in data = 5. Principal-component analysis was performed using TASSEL [66] and plotted using the PCA3D package in R.

### Phylogenetic tree

Evolutionary distances among the strains were computed using three methods; neighbor joining, maximum likelihood, and Euclidean hierarchical distance. The initial phylogenetic tree was obtained in FastME v2.0 using p-distance model and 1000 bootstrap replicates [67]. It was compared with a phylogenetic tree based on maximum likelihood method produced from the recoded alignments using RAxML [68] under the GTRGAMMA model and 1000 bootstrap replicates. Euclidean hierarchical distance was analyzed using selected subroutines in R; dendrograms were plotted using ggdendro, extended using dendextend, and the phylogenetic tree was plotted using ggplot2.

### Heat maps

Heat maps based on genome-wide SNPs were constructed using Euclidean hierarchical distance with centroid linkage as the clustering algorithm in ArrayStar.

### Nucleotide diversity and Tajima’s neutrality test

Nucleotide diversity (π) and Tajima’s neutrality test (D) were measured in sliding windows of 1 kb across the genome using VCFtools [64]. The average pairwise divergence within a clade (θπ) was estimated for the whole genome among various clades. A sliding window of 1 kb with 90% overlap between adjacent windows was used to estimate θπ for the whole genome.

### Linkage disequilibrium

Genome-wide pairwise linkage disequilibrium analysis (r^2^ and D’) was performed on all DNA variants using PLINK [69]. The average r^2^ value was calculated for each length of distance (< 1000 bp) and linkage disequilibrium decay was calculated and plotted using PopLDdecay.

## Supplementary information

**Additional file 1: Figure S1.**The ratio of homogenous to heterogeneous SNPs. A SNP frequency range of 25–75% was used; any SNP at lower than 75% is most likely a heterozygous SNP.

**Additional file 2: Figure S2.**Molecular phylogenetic analysis using the maximum likelihood method in RAxML. The percentage of 1000 bootstrap trials (1000 replicates) are shown above the branches. Strain details are summarized in their names: location by province (e.g., AB = Alberta), Williams’ pathotype (e.g., P3) where available, and SS = single-spore isolate, V = vegetable host, and C = canola host.

**Additional file 3: Figure S3.**Molecular phylogenetic analysis using the Euclidian hierarchical distance method from R packages. The height of the fusion, presented on the horizontal axis, indicates the dissimilarity between two strains. The larger the height of the fusion, the less similar the strains. Strain details are summarized in their names: location by province (e.g., AB = Alberta), Williams’ pathotype (e.g., P3) where available, and SS = single-spore isolate, V = vegetable host, and C = canola host.

**Additional file 4: Figure S4.** Nucleotide diversity across the genome of 43 strains of *Plasmodiophora brassicae*. The mean nucleotide diversity (θπ) was higher in Clades 1 and 3 (Clade 1 = 0.0011 and Clade 3 = 0.0011) compared with the other three clades (Clade 2 = 0.00097), Clade 4 = 0.00084, Clade 5 = 0.00084). The average θπ across all of the strains was 0.00095.

**Additional file 5. **Nucleotide diversity across the genome of 43 strains of *Plasmodiophora brassicae*.

**Additional file 6. ** Linkage disequilibrium (LD matrix) in 43 strains of *Plasmodiophora brassicae*.

**Additional file 7.** Number of variants per sample.

## Data Availability

The sequencing reads have been submitted and are available in NCBI BioProject repository (BioProject ID# PRJNA515478). Additional data sets supporting the results of this article are included within the article and its additional files.

## Abbreviations

MS: Murashige and Skoog medium
SNP: Single nucleotide polymorphism
SS: Single-spore isolate
θπ: Nucleotide diversity
