# Supplementary material for: Whole-genome DNA similarity and population structure of Plasmodiophora brassicae strains from Canada
Source: BMC Genomics. 2019 Oct 16;20:744. doi: 10.1186/s12864-019-6118-y (PMC6794840; doi:10.1186/s12864-019-6118-y)
Supplement: Supplementary file 1 — Additional file 1: Figure S1.The ratio of homogenous to heterogeneous SNPs. A SNP frequency range of 25–75% was used; any SNP at lower than 75% is most likely a heterozygous SNP. [file 12864_2019_6118_MOESM1_ESM.pptx]

## Slide 1
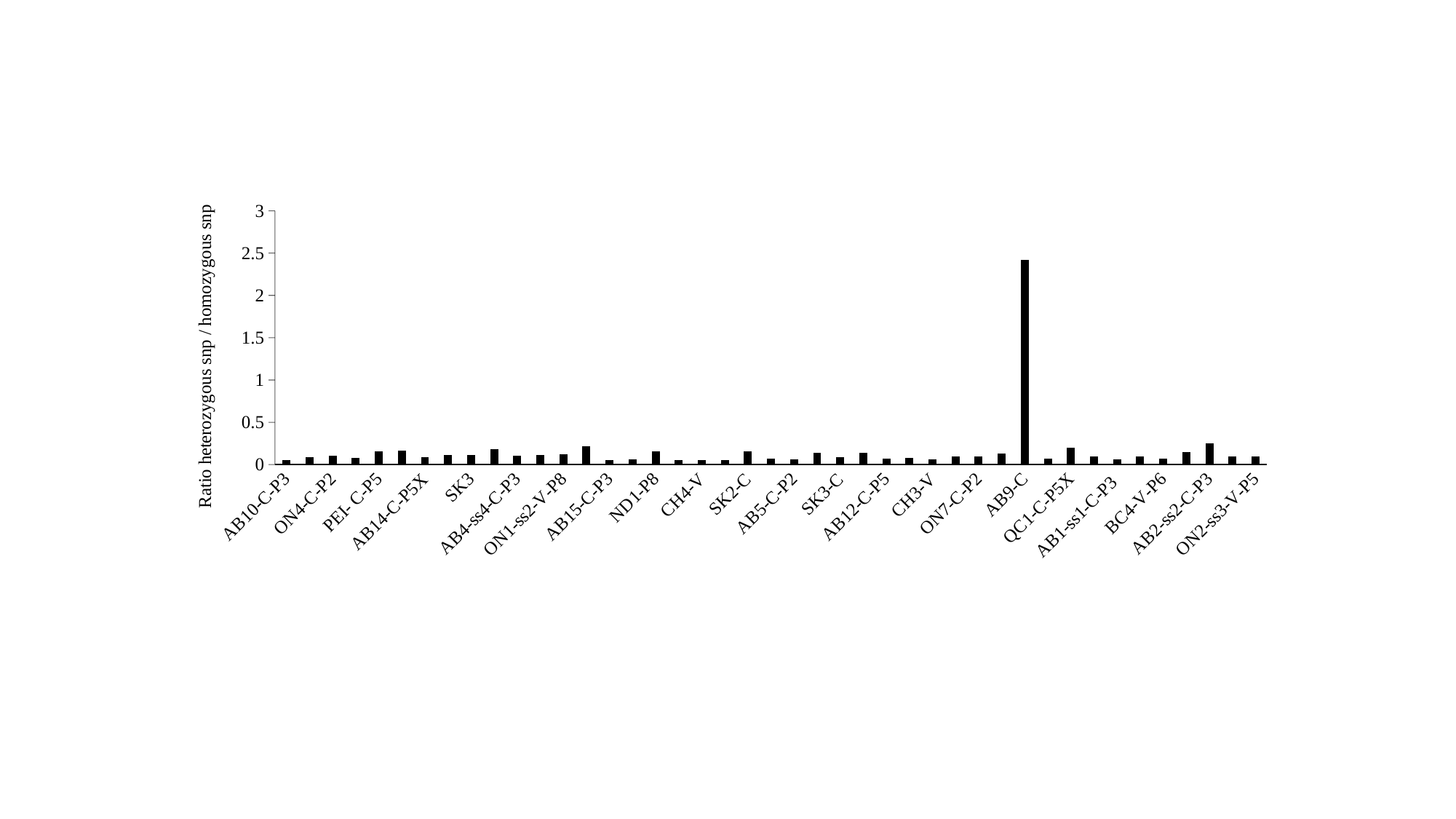

#
### Chart
| Category | Ratio heterozygous snp / homozygous snp |
|---|---|
| AB10-C-P3 | 0.0506828803484732 |
| ON5-C-P2 | 0.08671727479066929 |
| ON4-C-P2 | 0.0963896074682263 |
| ON6-C-P6 | 0.07356591717673205 |
| PEI- C-P5 | 0.15618437290263915 |
| ON3-V-P6 | 0.16119045023438353 |
| AB14-C-P5X | 0.08033292042464758 |
| AB13-C-P5X | 0.10553608969866854 |
| SK3 | 0.11083978658281624 |
| BC5-V-P6 | 0.1801229646645497 |
| AB4-ss4-C-P3 | 0.10455375167728444 |
| BC2-ss4-V-P6 | 0.10680583176054821 |
| ON1-ss2-V-P8 | 0.11961754704911962 |
| BC3-V-P6 | 0.2099549811211153 |
| AB15-C-P3 | 0.0530892495554482 |
| AB7-C | 0.05620296573841806 |
| ND1-P8 | 0.15221061517357815 |
| ND2-P8 | 0.05137659285670874 |
| CH4-V | 0.05008973731440692 |
| CH2-V | 0.04822081809112072 |
| SK2-C | 0.14818832718594055 |
| BC2-ss2-V-P6 | 0.06949708329018674 |
| AB5-C-P2 | 0.05462376689625057 |
| NF2-P1 | 0.1324966487284596 |
| SK3-C | 0.07918405192396848 |
| SK1-C-P3 | 0.13594297641082187 |
| AB12-C-P5 | 0.06489273521684767 |
| Ab11-C-P3 | 0.07136242884250474 |
| CH3-V | 0.05452615439051719 |
| CH5-V | 0.0910833665813571 |
| ON7-C-P2 | 0.0885256376124807 |
| NF1 | 0.12884911942952 |
| AB9-C | 2.411572925834067 |
| QC2-C-P2 | 0.06564664854930174 |
| QC1-C-P5X | 0.19863796133567663 |
| MB-C | 0.08965376384189822 |
| AB1-ss1-C-P3 | 0.059276085978184 |
| AB6-ss1-C-P8 | 0.08876085282638133 |
| BC4-V-P6 | 0.0693278575081987 |
| CH1-V-P1 | 0.14672767776506712 |
| AB2-ss2-C-P3 | 0.24460077915201028 |
| AB3-ss3-C-P2 | 0.0955886924163166 |
| ON2-ss3-V-P5 | 0.08985128599977507 |
