# Supplementary figures and images for: Whole-genome DNA similarity and population structure of Plasmodiophora brassicae strains from Canada
Source: BMC Genomics. 2019 Oct 16;20:744. doi: 10.1186/s12864-019-6118-y (PMC6794840; doi:10.1186/s12864-019-6118-y)

## Slide 1
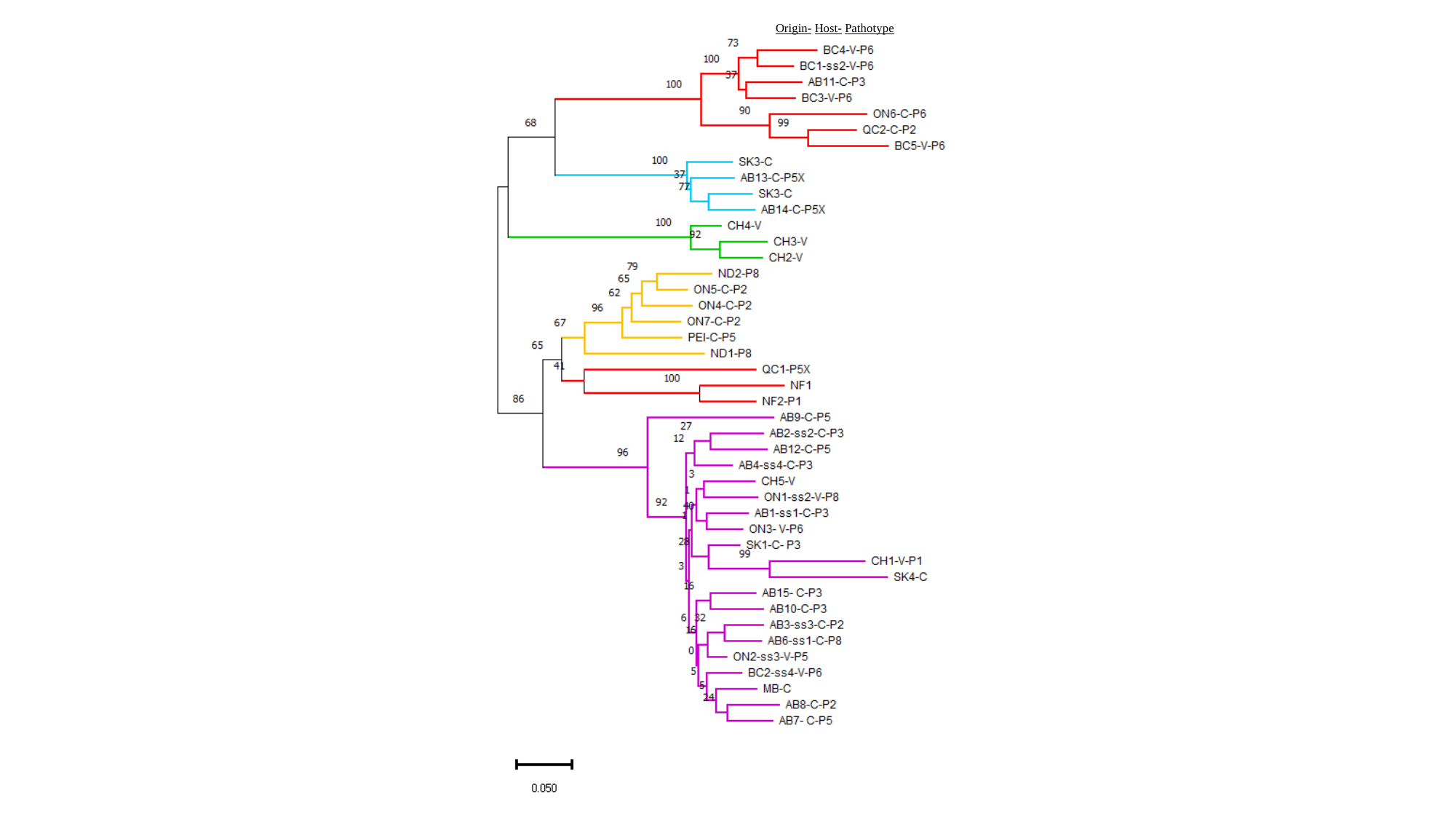

Origin- Host- Pathotype

Supplement: Supplementary file 2 — Additional file 2: Figure S2.Molecular phylogenetic analysis using the maximum likelihood method in RAxML. The percentage of 1000 bootstrap trials (1000 replicates) are shown above the branches. Strain details are summarized in their names: location by province (e.g., AB = Alberta), Williams’ pathotype (e.g., P3) where available, and SS = single-spore isolate, V = vegetable host, and C = canola host. [file 12864_2019_6118_MOESM2_ESM.pptx]

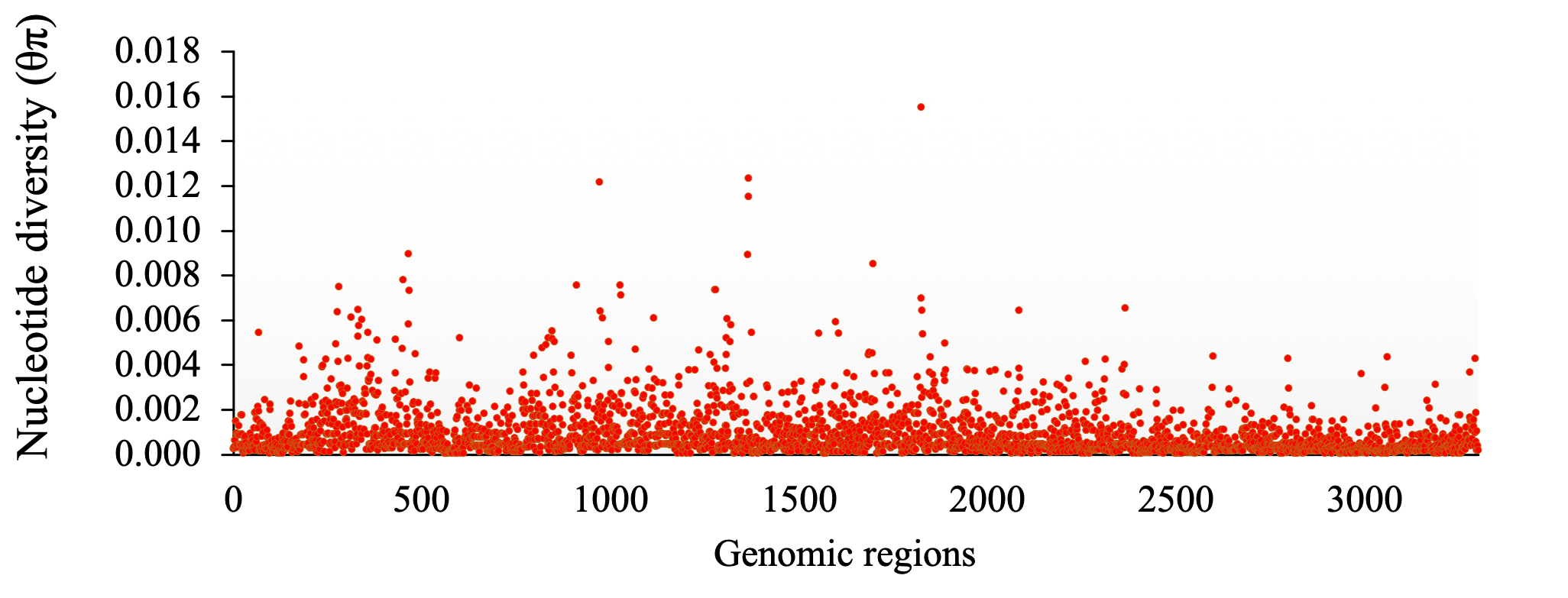

Supplement: Supplementary file 4 — Additional file 4: Figure S4. Nucleotide diversity across the genome of 43 strains of Plasmodiophora brassicae. The mean nucleotide diversity (θπ) was higher in Clades 1 and 3 (Clade 1 = 0.0011 and Clade 3 = 0.0011) compared with the other three clades (Clade 2 = 0.00097), Clade 4 = 0.00084, Clade 5 = 0.00084). The average θπ across all of the strains was 0.00095. [file 12864_2019_6118_MOESM4_ESM.png]
