# Supplementary material for: Whole-genome DNA similarity and population structure of Plasmodiophora brassicae strains from Canada
Source: BMC Genomics. 2019 Oct 16;20:744. doi: 10.1186/s12864-019-6118-y (PMC6794840; doi:10.1186/s12864-019-6118-y)
Supplement: Supplementary file 3 — Additional file 3: Figure S3.Molecular phylogenetic analysis using the Euclidian hierarchical distance method from R packages. The height of the fusion, presented on the horizontal axis, indicates the dissimilarity between two strains. The larger the height of the fusion, the less similar the strains. Strain details are summarized in their names: location by province (e.g., AB = Alberta), Williams’ pathotype (e.g., P3) where available, and SS = single-spore isolate, V = vegetable host, and C = canola host. [file 12864_2019_6118_MOESM3_ESM.pptx]

## Slide 1
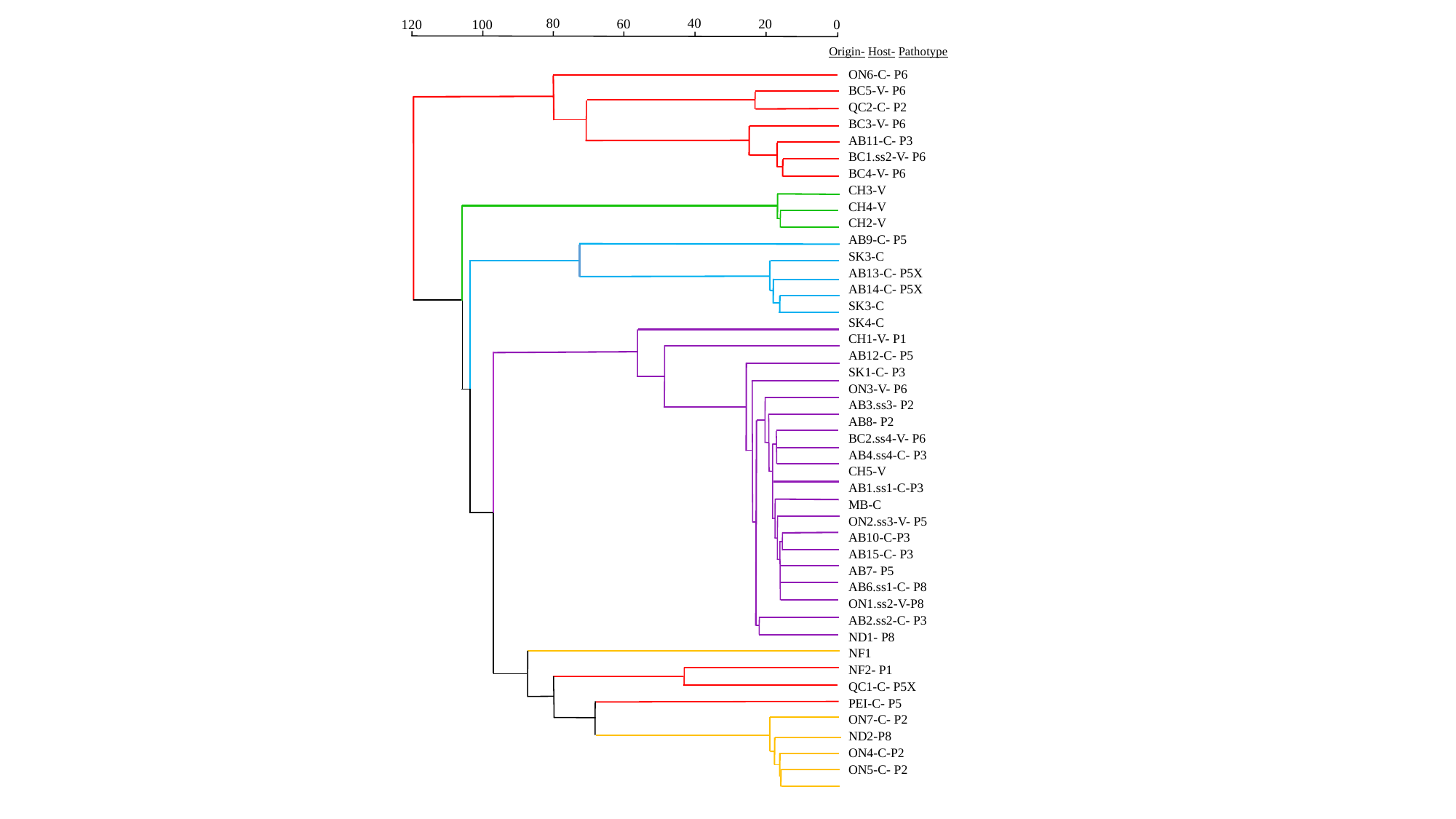

40
80
20
60
100
120
0
ON6-C- P6
BC5-V- P6
QC2-C- P2
BC3-V- P6
AB11-C- P3
BC1.ss2-V- P6
BC4-V- P6
CH3-V
CH4-V
CH2-V
AB9-C- P5
SK3-C
AB13-C- P5X
AB14-C- P5X
SK3-C
SK4-C
CH1-V- P1
AB12-C- P5
SK1-C- P3
ON3-V- P6
AB3.ss3- P2
AB8- P2
BC2.ss4-V- P6
AB4.ss4-C- P3
CH5-V
AB1.ss1-C-P3
MB-C
ON2.ss3-V- P5
AB10-C-P3
AB15-C- P3
AB7- P5
AB6.ss1-C- P8
ON1.ss2-V-P8
AB2.ss2-C- P3
ND1- P8
NF1
NF2- P1
QC1-C- P5X
PEI-C- P5
ON7-C- P2
ND2-P8
ON4-C-P2
ON5-C- P2
Origin- Host- Pathotype
